# Growth and growth trajectory among infants in early life: contributions of food insecurity and water insecurity in rural Zimbabwe

**DOI:** 10.1136/bmjnph-2022-000470

**Published:** 2022-11-30

**Authors:** Nadia Koyratty, Robert Ntozini, Mduduzi NN Mbuya, Andrew D Jones, Roseanne C Schuster, Katarzyna Kordas, Chin-Shang Li, Naume V Tavengwa, Florence D Majo, Jean Humphrey, Laura E Smith

**Affiliations:** 1 Department of Poverty, Health and Nutrition, International Food Policy Research Institute, Washington DC, Washington DC, USA; 2 Statistics, Zvitambo Institute for Maternal and Child Health Research, Harare, Zimbabwe; 3 Knowledge Leadership, Global Alliance for Improved Nutrition, Geneva, Switzerland; 4 Department of Nutritional Sciences, University of Michigan, Ann Arbor, Michigan, USA; 5 School of Human Evolution and Social Change, Arizona State University, Tempe, Arizona, USA; 6 Department of Epidemiology and Environmental Health, University at Buffalo, Buffalo, New York, USA; 7 School of Nursing, University at Buffalo, Buffalo, NY, USA; 8 Department of International Health, Johns Hopkins Bloomberg School of Public Health, Baltimore, Maryland, USA; 9 Department of Public and Ecosystem Health, Cornell University, Ithaca, New York, USA

**Keywords:** Malnutrition

## Abstract

**Introduction:**

Stunting or linear growth faltering, measured by length-for-age Z-score (LAZ), remains a significant public health challenge, particularly in rural low-income and middle-income countries. It is a marker of inadequate environments in which infants are born and raised. However, the contributions of household resource insecurities, such as food and water, to growth and growth trajectory are understudied.

**Methods:**

We used the cluster-randomised Sanitation Hygiene and Infant Nutrition Efficacy trial to determine the association of household-level food insecurity (FI) and water insecurity (WI) on LAZ and LAZ trajectory among infants during early life. Dimensions of FI (poor access, household shocks, low availability and quality) and WI (poor access, poor quality, low reliability) were assessed with the multidimensional household food insecurity and the multidimensional household water insecurity measures. Infant length was converted to LAZ based on the 2006 WHO Child Growth Standards. We report the FI and WI fixed effects from multivariable growth curve models with repeated measures of LAZ at 1, 3, 6, 12 and 18 months (M1–M18).

**Results:**

A total of 714 and 710 infants were included in our analyses of LAZ from M1 to M18 and M6 to M18, respectively. Mean LAZ values at each time indicated worsening linear growth. From M1 to M18, low food availability and quality was associated with lower LAZ (β=−0.09; 95% −0.19 to –0.13). From M6 to M18, poor food access was associated with lower LAZ (β=−0.11; 95% −0.20 to –0.03). None of the WI dimensions were associated with LAZ, nor with LAZ trajectory over time.

**Conclusion:**

FI, but not WI, was associated with poor linear growth among rural Zimbabwean infants. Specifically, low food availability and quality and poor food access was associated with lower LAZ. There is no evidence of an effect of FI or WI on LAZ trajectory.

**WHAT IS ALREADY KNOWN ON THIS TOPIC**

- Food insecurity, in the form of inadequate food access, is an underlying risk factor for stunting and poor child growth.
- Contaminated drinking water is also a known risk factor for poor child growth and health.

**WHAT THIS STUDY ADDS**

- Different aspects of food insecurity (availability, quality, access) are associated with lower length-for-age Z-score (LAZ).
- Poor water access, poor water quality and low water reliability are not associated with LAZ.
- Neither food insecurity nor water insecurity is associated with infant growth trajectory.

**HOW THIS STUDY MIGHT AFFECT RESEARCH, PRACTICE OR POLICY**

- For improved child growth, renewed attention should be paid to improving food availability and quality, in addition to ensuring adequate food access.
- Further research is needed to investigate the link between water insecurity conceptualised broadly and child growth.

## Introduction

Stunting represents the most prevalent form of child malnutrition globally, with a 2020 estimate of 144 million children under 5 years of age affected.^1^ Stunted growth occurs when infant length or height falls below negative 2 SDs(<−2 SDs), in comparison with the WHO Child Growth Standards for the same age and sex group.^2^ Several more millions of children suffer from less severe forms of growth faltering,^3^ and any amount of poor linear growth adversely affects child health and well-being.^3 4^

Linear growth faltering often begins at conception and continues for about 2 years after the child’s birth,^5^ beyond which time, it is considered largely irreversible.^6^ Children with poor linear growth are more likely to experience higher rates of morbidity,^7 8^ ^7,8^ mortality,^9^ suboptimal cognitive, motor and social development,^10–12^ and later incidence of chronic diseases such as obesity and other cardiometabolic diseases during adulthood.^13 14^ Growth faltering also has intergenerational effects, whereby women who were stunted during childhood are at greater risk of bearing stunted children.^5^ As a marker for these distal negative outcomes on child health and well-being, stunting represents a valuable measure of public health importance.^15^

The UNICEF Conceptual Framework for Undernutrition^16^ and its adapted versions^8 17–19^ identify a host of risk factors for linear growth faltering at multiple levels. One key underlying determinant is household-level food insecurity (FI), defined as the opposite of when ‘all people at all times have physical, social and economic access to sufficient, safe and nutritious food that meets their dietary needs and food preferences for an active and healthy life’.^20^ Epidemiological evidence on the association between household FI and linear growth of children under 5 is inconclusive, varying from positive,^21–28^ to no association,^29–32^ and even to mixed associations.^33 34^ These existing studies have focused primarily on poor food access, experiences associated with inadequate food access, or diversity of household diet, as measures of FI. Other aspects of FI such as availability, utilisation and stability of the food supply have not been investigated. Moreover, with the exception of one longitudinal study in rural Bangladesh with infants followed from birth to 24 months,^21^ all studies have assessed the relationship between FI and infant length at a single time point. Therefore, growth and growth trajectory during early life remain unclear in relation to household FI.

The second major household driver of undernutrition suggested by the UNICEF framework relates to water insecurity (WI), specifically unsafe water. Contaminated water is assumed to compromise infant growth by increasing the risk of diarrhoeal diseases via poor sanitation and hygiene.^35–37^ The current literature on the association between water and linear growth is inconsistent, perhaps due to the narrow definition of water insecurity as untreated water^38–40^ or non-piped sources^41–44^ But household-level WI, such as FI, includes dimensions beyond unsafe water such as availability or sufficiency, access, affordability and reliability of the water supply in order to support well-being and the capacity to undertake economic, social and cultural activities.^45–47^ For example, WI assessment should consider water for consumption (eg, drinking, cooking), non-drinking hygiene (eg, bathing, cleaning) and subsistence (eg, agriculture, animal care). These WI dimensions may impact growth and growth trajectory by influencing maternal and infant hydration and health, food availability and preparation, and infant feeding behaviours.^48–51^ Recent nationally representative cross-sectional data employing a broader conceptualisation inclusive of household and regional WI identified an indirect effect of dietary diversity on stunting, but causal evidence is lacking.^52^ Thus, the limited consideration given to WI constitutes an important gap in the current literature on stunting.

The burden of growth faltering is highly prevalent in South Asia and sub-Saharan Africa (SSA),^1 53^ where the majority of the world’s 750 million severely food insecure^1^ and two billion water insecure people live.^54^ The focus of our study is the rural population of Zimbabwe, a land-locked country in SSA. Due to the political situation, complicated financial and trade situations, and erratic climate, Zimbabweans are highly affected by both FI and WI.^55^ Furthermore, about one-third of infants in rural Zimbabwe are stunted,^56^ and many more experience some level of linear growth faltering.^57 58^ Yet, the contributions of critical household resources, like food and water, on infant growth have not been properly examined. In countries such as Zimbabwe, addressing FI and WI, depending on the type of intervention, may also have long-term impacts that go beyond the improvement of child growth for example, economic development, women empowerment, increase in school attendance and social advancement.

The Sanitation Hygiene and Infant Nutrition Efficacy (SHINE) trial carried out in rural Zimbabwe reported improved length-for-age Z-score (LAZ) (β= +0.16; 95% CI 0.08 to 0.23) and reduced stunting (RR=0.80; 95% CI 0.73 to 0.88) among infants aged 18 months who received a nutrition intervention consisting of infant nutrient supplementation and maternal nutrition counselling,^59^ compared with those who did not. These improvements, although significant and consistent with other similar studies,^39 40^ are quite modest. A higher rate of stunting decline is required if Zimbabwe is to meet the World Health Assembly nutrition target of 40% reduction by 2025.^60^ Given the gaps identified above, we proposed that resource insecurities of households in which children are born and raised prevent higher effectiveness of current nutrition interventions. This is because resource insecurities prevent adequate child caring and feeding practices.^61^ Therefore, the purpose of our study was to determine the associations between different aspects of household-level FI and WI on infant growth during the first 1000 days of life. We hypothesised that higher levels of FI and WI will be associated with greater growth faltering and deteriorating trajectories of growth among infants under 2 years of age.

## Methodology

### Study design

The SHINE trial design and primary outcomes have been published previously.^59 62^ Additional information on the protocol and statistical analysis plan are available elsewhere (https://osf.io/w93hy). In summary, SHINE randomly assigned clusters, in two rural Zimbabwean districts (Shurugwi and Chirumanzu), to receive one of four interventions: (1) standard of care (SOC), (2) infant and young child feeding (IYCF), (3) water, sanitation, hygiene (WASH) and (4) IYCF+WASH. The clusters were defined as the catchment area of 1–4 village health workers employed by the Ministry of Health and Child Care. Between 22 November 2012 and 27 March 2015, pregnant women aged 15–49 years old who were permanent residents of those rural areas were enrolled. The infants born to the pregnant women were followed over time to ascertain stunting and anaemia at M18. The analyses presented in this paper focus on the SOC arm (n=1166 live born infants), which received only the WHO recommended education modules on optimal breastfeeding practices for all infants from birth to M6. Thus, the SOC arm was considered appropriate for investigating the effects of FI and WI on infant growth and growth trajectory, independent of the SHINE interventions.

### Data collection

Research nurses made home visits at multiple times to collect relevant information from households, mothers and infants: at baseline (during pregnancy) and at infant ages 1, 3, 6, 12 and 18 months (M1–M18).

Growth: We used LAZ as the indicator for growth. Recumbent length was measured to the nearest 0.1 cm using a Seca 417 infantometer by trained nurses. The length measurements at each time were converted to LAZ based on the 2006 WHO Child Growth Standards.^63^

FI and WI: The multidimensional household food insecurity (MHFI) and the multidimensional household waterinsecurity (MHWI) measures, developed specifically for the rural Zimbabwean households, were used.^64^ These measures were created from separate factor analyses using groups of food-related and water-related variables collected at baseline (during pregnancy) from the SHINE trial. From these analyses, FI and WI were characterised by three dimensions each. MHFI includes (1) poor food access, (2) household shocks and (3) low food availability and quality; whereas MHWI includes (1) poor water access, (2) poor water quality and (3) low water reliability. A description of the variables making up each dimension is provided in table 1. Each MHFI and MHWI dimension was scored in postestimation commands in the ‘PCAmix’ package from the R software (R Foundation for Statistical Computing, Vienna, Austria) V.4.0.2. We used each of these three dimensions of FI and WI as the main continuous exposure variables in this study. These variables were included simultaneously in the statistical models. An important note is that higher scores on the dimensions of FI and WI as described in table 1 represent higher levels of insecurity.

**Table 1:** Description of MHFI and water insecurity

Insecurity	Dimension	Variables used for scoring
MHFI	Poor food access	Preferred food (yes/no) Sufficient food (yes/no) Help to get food (yes/no) Food on credit (yes, no)
	Household shocks	Agriculture (yes/no) Economic losses (yes/no) Death or injury (yes/no)
	Low availability and quality	Stock of staple food (8 ordinal categories of time) Having a home garden (yes/no) Diet diversity of household (yes/no)
MHWI	Poor water access	Distance (>1000 m) Time to drinking water (>15 min) Time to non-drinking water (>15 min)
	Poor water quality	Type of drinking source(improved (piped, protected ground), unimproved ground (unprotected boreholes/wells), surface) Type of non-drinking source(improved (piped, protected ground), unimproved ground (unprotected boreholes/wells), surface) Drinking water satisfaction related to taste, smell and colour (satisfied, neutral, unsatisfied)
	Low water reliability	Unavailability of water for drinking (ever/never in the past year) Unavailability of non-drinking purposes (ever/never in the past year)

MHFI, multidimensional household food insecurity; MHWI, multidimensional household water insecurity.

Covariates: At baseline (during pregnancy), a structured questionnaire was used to collect information on maternal and household characteristics such as maternal age (years), maternal height (cm), maternal education (some primary, some secondary, completed secondary), formal employment outside the home (yes/ no), marital status (married vs other), religion (apostolic, other Christian, other), parity (parous, nulliparous, missing), household size (number of household members), presence of improved latrine (yes/no), household location (Shurugwi/Chirumanzu) and season at baseline (during pregnancy) interview (calendar quarter). The HIV status of women was determined using a rapid test algorithm; those who tested positive were directed to local clinics for follow-up and treatment. Socioeconomic status (SES) was based on a household wealth index created specifically for this population.^65^ Maternal depression, based on Edinburgh’s Postnatal Depression Scale,^66^ and mothering self-efficacy^67^ were collected using validated scales for the Zimbabwean population as described previously. Pregnant women’s diet adequacy was assessed based on food group consumption, as described in the FANTA project Minimum Dietary Diversity for Women (yes/ no).^68^ Infant characteristics such as date of birth, sex, birth weight and prematurity (born at<37 weeks of gestation) were abstracted from health facility records. Infant breast feeding in the 24 hours prior to interviews at M6, M12 and M18 was self-reported by the mother.

Since SHINE was household based, the intermediate visits were conducted only when mother–infant dyads still lived at the address where they consented. If after two contact attempts the participants remained inaccessible, they were considered missing at those time points. At M18, participants were visited anywhere in Zimbabwe even if they had moved on from their initial residence. In addition to our sample being restricted to infants from the SOC arm, analyses were further limited to infants who had complete information on FI, WI, at least one LAZ measure out of five and the above prespecified covariates. Infants, who had died prior to the end of the trial (n=67), whose mothers signed voluntary consent to exit the study (n=5) and who had implausible LAZ patterns over time, were also excluded (n=3).

### Statistical analyses

Descriptive statistics were used to summarise the characteristics of the infants included in the analysis. Frequencies and percentages were used for categorical variables. Medians (p50) and IQRs were used for the distributions of the FI and WI dimensions. After graphically confirming normal LAZ distribution of our sample, LAZ values were summarised using means and SDs. The associations of FI and WI with LAZ and LAZ trajectory were investigated through multivariable growth curve modelling of their fixed effects. We used unstructured covariance structure to account for multiple measurements of length on the same infant over time. Time interactions with FI and WI represented growth trajectory associated with these exposures in our models.

Two groups of variables were defined a priori. Group 1 included only variables that were considered theoretically critical given the main predictors and population: season at baseline (during pregnancy) interview, household SES, infant sex, residence location, improved latrine and maternal HIV status. Group 2 additionally included risk factors for poor growth: maternal age, height, education, religion, parity, maternal depression, mothering self-efficacy, infant birth weight, prematurity, breast feeding and household size. Group 2 variables and time-covariate interactions were selected using backward stepwise regressions with retention at p<0.2 at each modelling stage. Multicollinearity was tested with variance inflation factors (VIF <5). The best subset of covariates for the growth models was identified by comparing AIC and BIC between models. Two models are presented in the results section. Model 1 consists of group 1 variables and time interaction with infant sex (minimally adjusted model (Min-AM)). Model 2 includes Min-AM, plus maternal age, height, education, religion, infant birth weight, preterm birth, household size, continued breast feeding until M18 and time interactions with maternal height, infant birth weight and continued breast feeding until M18 (fully AM (Full-AM)). All analyses were performed in Stata/MP V.17 (StataCorp).

### Patient and public involvement

Patients or the public were not involved in the design, or conduct, or reporting, or dissemination plans of the research presented in this paper.

## Results

### Population characteristics

**Figure 1:**
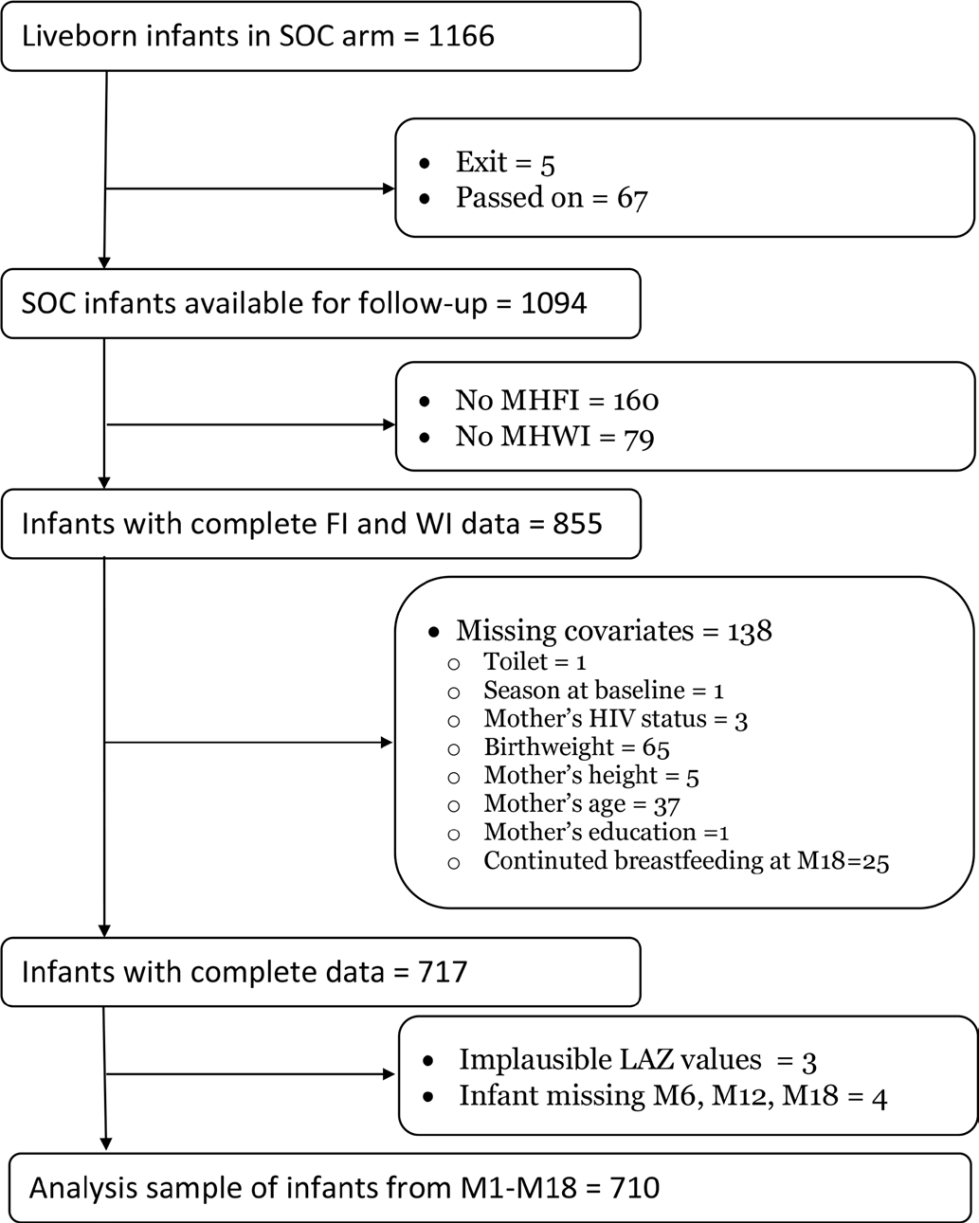
Selection of infants for inclusion in growth analyses. LAZ: Lengthforage Zscore, SOC= Standard of Care; FI= food insecurity; WI= water insecurity; MHFI= Multidimensional Household Food Insecurity; MHWI= Multidimensional Household Water Insecurity; M1, M3, M6, M12, M18= 1, 3, 6, 12, 18 months.

Of the 1166 infants born to women enrolled in SHINE’s SOC arm, 714 and 710 infants had complete information for growth analysis from M1 to M18 and from M6 to M18 (figure 1). Table 2 describes the characteristics of those infants, their mothers and households. Follow-up of the mother–infant dyads was lowest at M1 and M3 study visits because Zimbabwean women typically move back to their maternal home in the immediate postnatal period. On average, infants weighed 3.08 (±0.48) kg at birth, although 19.9% were born preterm. More than half of the infants were still breastfed at M18 (54.3%). Mean LAZ values at M1, M3 and M6 were similar and did not indicate stunting for the sample, although lower values were observed at M12 (−1.27±1.09) and M18 (−1.60±1.09). Dimension scores of FI and WI were below zero, suggesting modest overall FI and WI levels.

**Table 2:** Description of infants, their caregivers and households included in analyses

Characteristics	M1–M18	M6–M18
n	714	710
Infants		
Female, n (%)	346 (48.5)	345 (48.6)
Preterm, n (%)	141 (19.8)	138 (19.4)
Low birth weight, n (%)	67 (9.4)	65 (9.2)
Birth weight (kg), n/mean (SD)	714/3.08 (0.48)	710/3.08 (0.48)
Small for gestational age, n (%)	67 (12.8)	66 (12.7)
Length-for-age Z score, n/mean (SD)		
Month 1	446/−0.98 (1.4)	443/−0.96 (1.38)
Month 3	463/−0.89 (1.23)	462/−0.89 (1.22)
Month 6	534/−0.95 (1.2)	534/−0.95 (1.2)
Month 12	559/−1.27 (1.09)	559/−1.27 (1.09)
Month 18	707/−1.60 (1.09)	707/−1.60 (1.09)
Stunting		
Month 1	446/88 (19.7)	443/85 (19.2)
Month 3	463/72 (15.6)	462/71 (15.4)
Month 6	534/97 (18.2)	534/97 (18.2)
Month 12	559/143 (25.6)	559/143 (25.6)
Month 18	707/252 (35.6)	707/252 (35.6)
Age at interview (days), n/mean (SD)		
Month 1	447/44.05 (16.52)	443/44.11 (16.56)
Month 3	465/109.69 (22.64)	464/109.71 (22.66)
Month 6	540/214.44 (47.96)	540/214.44 (47.96)
Month 12	559/388.82 (42.25)	559/388.82 (42.25)
Month 18	709/554.61 (46.92)	707/554.63 (46.97)
Breastfed at M6, n (%)		
No	6 (0.8)	6 (0.9)
Yes	516 (72.3)	514 (72.4)
Missing	192 (26.9)	190 (26.8)
Breastfed at M12, n (%)		
No	27 (3.8)	27 (3.8)
Yes	539 (75.5)	537 (75.6)
Missing	148 (20.7)	146 (20.6)
Breastfed at M18, n (%)		
No	326 (45.7)	324 (45.6)
Yes	388 (54.3)	386 (54.4)
Mothers		
Age (years), n/mean (SD)	714/26.4 (6.6)	710/26.4 (6.6)
Height (cm), n/mean (SD)	714/160.38 (6.49)	710/160.45 (6.33)
Education, n (%)		
Some primary	123 (17.2)	120 (16.9)
Some secondary	262 (36.7)	262 (36.9)
Completed secondary	329 (46.1)	328 (46.2)
Married, n (%)	675 (94.5)	671 (94.5)
Employed outside home*, n (%)	45 (6.3)	45 (6.3)
Parity, n (%)		
Parous	439 (61.5)	437 (61.6)
Nulliparous	86 (12.0)	85 (12.0)
Missing	189 (26.5)	188 (26.5)
HIV negative, n (%)	613 (85.9)	609 (85.8)
Religion, n (%)		
Apostolic	347 (48.6)	344 (48.5)
Other Christian	320 (44.8)	319 (44.9)
Other religion	47 (6.6)	47 (6.6)
Depression score*†, n/median (IQR)	701/1 (4)	697/1 (4)
Mothering self-efficacy*‡, n/mean (SD)	699/4.0 (0.42)	695/4.0 (0.42)
Meet diet diversity*, n (%)	127 (18.0)	127 (18.1)
Interview at baseline (pregnancy period), n (%)		
January–March	255 (35.7)	254 (35.8)
April–June	154 (21.6)	153 (21.6)
July–September	149 (20.9)	148 (20.9)
October–December	156 (21.9)	155 (21.8)
Households		
No of members, n/median (IQR)	714/5 (3)	710/5 (3)
SES, n (%)		
Lower	248 (34.7)	246 (34.7)
Middle	229 (32.1)	229 (32.3)
Upper	237 (33.2)	235 (33.1)
Improved latrine, n (%)	237 (33.2)	236 (33.2)
Residence, n (%)		
Chirumanzu	335 (46.9)	332 (46.8)
Shurugwi	379 (53.1)	378 (53.2)
MHFI§, n/median (IQR)		
Poor food access	714/−0.54 (1.08)	710/−0.54 (1.08)
Household shocks	714/−0.08 (1.16)	710/−0.08 (1.16)
Low food availability and quality	714/−0.12 (1.50)	710/−0.12 (1.50)
MHWI§, n/median (IQR)		
Poor water access	714/−0.49 (1.38)	710/−0.49 (1.38)
Poor water quality	714/−0.44 (2.10)	710/−0.44 (2.10)
Low water reliability	714/−0.35 (0.29)	710/−0.35 (0.29)

All variables were reported or measured at baseline (during pregnancy) unless otherwise stated.

*Variables with <5% missing.

†Measured using the Edinburgh Postnatal Depression Scale (score: 0–30).

‡Mothering self-efficacy measured using questions adapted from the Parenting Sense of Competence Scale and Parenting Self-Agency Measure (score: 0–5).

§Higher scores on dimensions of MHFI and MHWI indicate higher insecurity.

MHFI, multidimensional household food insecurity; MHWI, multidimensional household water insecurity; SES, socioeconomic status.

### LAZ, LAZ trajectory and FI

Of the three FI dimensions investigated, the dimension of ‘low food availability & quality’ was the only one associated with LAZ from M1 to M18 in both growth curve models (table 3): β_MIN-AM_= −0.09; 95% CI −0.17 to –0.01 and β_FULL-AM_= −0.09; 95% CI −0.17 to –0.01. However, there was no association between any FI dimensions and M1-M18 LAZ trajectory. In growth models from M6 to M18, poor food access (β_MIN-AM_= −0.12; 95% CI −0.21 to –0.03; β_FULL-AM_= −0.11; 95% CI −0.20 to –0.03) was associated with lower LAZ, but LAZ trajectory was still not associated with any FI dimension. In post hoc analysis where we explored length instead of LAZ, we found an association between the low food availability and quality dimension and M6–M18 length trajectory (β_MIN-AM_= −0.03; 95% CI −0.05 to –0.01; online supplemental table S1). However, after adjusting for time interactions with continued breastfeeding until M18, maternal height, infant sex and infant birth weight the association was no longer significant (β_FULL-AM_= −0.02; 95% CI −0.04 to 0.00).

10.1136/bmjnph-2022-000470.supp1 Supplementary data

**Table 3:** Association of infant LAZ and LAZ trajectory with food insecurity and water insecurity

	M1–M18 (n=714)		M6–M18 (n=710)	
Model	Min-AM	Full-AM	Min-AM	Full-AM
LAZ				
Food insecurity				
Poor food access	−0.04 (−0.13, 0.05)	−0.03 (−0.1, 0.05)	−**0.12 (−0.21, 0.03**)	−**0.11 (−0.2, 0.03**)
Household shocks	−0.01 (−0.1, 0.07)	−0.02 (−0.09, 0.06)	0 (−0.1, 0.09)	0 (−0.08, 0.09)
Low food availability & quality	−**0.09 (−0.18, 0.01**)	−**0.09 (−0.17, 0.01**)	−0.07 (−0.16, 0.02)	−0.06 (−0.14, 0.03)
Water insecurity				
Poor water access	−0.02 (−0.11, 0.07)	0 (−0.08, 0.07)	−0.02 (−0.11, 0.07)	−0.02 (−0.10, 0.07)
Poor water quality	0.04 (−0.05, 0.12)	0 (−0.08, 0.07)	0.08 (−0.01, 0.16)	0.04 (−0.04, 0.12)
Low water reliability	0.06 (−0.03, 0.14)	0.03 (−0.05, 0.1)	0.05 (−0.04, 0.14)	0.03 (−0.05, 0.12)
LAZ trajectory				
Time	−**0.04 (−0.04, 0.03**)	−0.03 (−0.1, 0.05)	−**0.05 (−0.06, 0.04**)	−0.13 (−0.28, 0.03)
Timexfood insecurity				
Poor food access	0 (−0.01, 0)	0 (−0.01, 0)	0 (0, 0.01)	0 (0, 0.01)
Household shocks	0 (0, 0.01)	0 (0, 0.01)	0 (0, 0.01)	0 (0, 0.01)
Low food availability and quality	0 (−0.01, 0)	0 (−0.01, 0)	0 (−0.01, 0)	0 (−0.01, 0)
Timexwater insecurity				
Poor water access	0 (−0.01, 0)	0 (−0.01, 0)	0 (−0.01, 0)	0 (−0.01, 0)
Poor water quality	0 (0, 0.01)	0 (0, 0.01)	0 (−0.01, 0.01)	0 (−0.01, 0.01)
Low water reliability	0 (−0.01, 0)	0 (−0.01, 0)	0 (−0.01, 0)	0 (−0.01, 0)
Model diagnostics				
AIC	7609.732	7262.259	4727.371	4553.204
BIC	7763.244	7492.528	4870.255	4767.53

Min-AM: season at baseline (during pregnancy) interview (calendar quarter), SES (tertile), infant sex (female vs male), household location (Chirumanzu vs Shurugwi), improved latrine (no vs yes) and maternal pregnancy HIV status (positive vs negative), time–sex interaction.

Full-AM: Min-AM+maternal age (years), height (cm), education (some primary, some secondary, completed secondary), religion (Apostolic, other Christian, other religion), infant birthweight (kg), preterm (born at <37 weeks of gestation: yes vs no), household size (number of members), continued breastfeeding until M18 (yes vs no), and time interactions with maternal height, infant birthweight and continued breastfeeding until M18.

Values in bold signifies P<0.05.

AIC, Akaike information Criterion; BIC, Bayesian information criterion; Full-AM, fully-AM; LAZ, length-for-age Z-score; Min-AM, minimally adjusted model; SES, socioeconomic status.

### LAZ, LAZ trajectory and WI

None of the WI dimensions were associated with LAZ or LAZ trajectory from M1 to M18, nor from M6 to M18 (table 3).

## Discussion

This study examined the associations of household FI and WI on linear growth and growth trajectory of infants from 1 to 18 months of age in rural Zimbabwe. We found evidence that FI, but not WI, is negatively associated with LAZ among these infants. We found no associations of FI or WI with LAZ trajectory (or growth velocity), quantified via time interactions with FI and WI.

### Growth, growth trajectory and FI

Two FI dimensions were associated with growth faltering: low food availability and quality (M1–M18), and poor food access (M6–M18). We found no relationship between FI and growth trajectory. These FI results are consistent with some prior literature.

Among cross-sectional studies of the association between food (in)security and LAZ, some show no association,^30–34 69^ while others show lower LAZ or stunting prevalence.^23–26 28 29 34^ To our knowledge, only one other longitudinal study investigated the relationship between food security and indicators of infant growth and growth trajectory.^21^ The Maternal and Infant Nutrition Intervention in Matlab study in rural Bangladesh enrolled women during their pregnancy and followed their infants monthly from one to 24 months after birth.^21^ Consistent with our results, it found a trend for higher LAZ values with increasing household food security levels, categorised into quartiles (p<0.01). However, the LAZ trajectory remained the same across all four categories of food security.

Contrary to our hypothesis, we found no evidence for an association between FI and LAZ trajectory. This finding suggests that during the first 18 months after birth, FI did not contribute to the deterioration in LAZ trajectory over time among Zimbabwean infants. Nevertheless, this does not mean that absolute length deficits do not worsen with increasing FI. For instance, in the above-mentioned study among Bangladeshi infants, the multilevel models for change showed that infants from food secure households were on average 0.08 cm taller, and had a better growth trajectory based on absolute increase in length (+0.01 cm per month), compared with food insecure infants in that population.^21^ It has been suggested that inconsistencies in growth findings across studies may be due to the parameterisation of the anthropometric measures. It has been recommended that absolute length-for-age differences (ie, differences between two time points), rather than LAZ or length, may be more efficient at quantifying growth trajectories.^70^ However, others suggest divergent approaches with different statistical methods and anthropometric parameterisation to better capture the growth faltering phenomenon.^71^ It is also worth mentioning that households, in the event of FI, may employ certain behaviours to ensure that the children are fed. In post hoc analyses, households’ who were insecure for the three FI dimensions reported coping behaviours such as hunting or gathering wild foods, harvesting green maize, sending children to eat elsewhere, and reducing adult consumption so that there is sufficient food for children. These practices may explain the lack of association between some of the dimensions of FI and LAZ or LAZ trajectory.

Household FI could influence infant growth in multiple ways. As indicated by the FI dimensions identified in our study, different aspects of FI may be important for growth at various times and even in different settings. For example, the low food availability and quality dimension was made up of variables related to quantity of staple food in stock for the household, whether the household had a garden, and whether the household members were able to eat diverse foods; whereas the poor food access dimension was made up of food insufficiency, unavailability of preferred food and insufficient resources to obtain food. Households without a garden are less likely to have infants meeting their nutrient adequacy, which can negatively influence growth.^72^ The FI experiences of not having sufficient quantities of food, resources to obtain food, and in rural areas, not having staple foods stored for the lean season, may be associated with poor maternal nutritional status,^73–75^ maternal depressive symptoms,^76–79^ feelings of inadequacy of mothering ability^80^ and intimate partner violence.^81 82^ These factors may contribute to poor parenting, caring and infant feeding practices,^67 83–86^ which potentially mediate the association from FI to poor infant growth.^87–89^

Our results may not be directly comparable to other studies because our FI indicators are different from what is commonly used for assessments. First, we have used distinct indicators for separate FI dimensions, while most previous studies have used experience-based and unidimensional measures of FI such as HFIAS or HDDS.^90^ Second, some studies have only found associations between severe FI and growth, but not at low or mild FI levels.^23 32 33 91^ Third, the choice of growth parameterisation varies from continuous LAZ, stunting or severity of stunting. Fourth, as discussed above, our longitudinal findings may not be directly compared with the existing cross-sectional studies that mostly account for anthropometric measurement at a single time point. Fifth, the age range of infants in the available literature is primarily from 6 months to 5 years old, while our infant sample was followed from 1 month to 18 months, which is within the critical period of 1000 days of life.

### Growth, growth trajectory and WI

We found no evidence of associations between dimensions of household WI and infant growth or growth trajectory from 1 to 18 months of age. This was contrary to our hypothesis that poor water access, poor water quality and low water reliability would be associated with lower LAZ and deteriorating LAZ trajectory.

Traditionally, the contribution of water to infant growth faltering has been investigated in relation to the increased risk of diarrhoeal diseases, and linked to poor sanitation and hygiene.^37 92^ Both observational and experimental studies have investigated the association between various indicators of water quality and growth faltering, with mixed results. For instance, cluster-randomised trials in Bangladesh^39^ and in Kenya^40^ both concluded that their household drinking water treatment interventions with chlorine had no effect on LAZ at the end of 2 years of follow-up. Conversely, Demographic Health Survey data from 70 LMICs showed that compared with surface water used as drinking source, high quality (piped or bottled; OR 0.92, 95% CI 0.89 to 0.94) and intermediate quality (boreholes or wells; OR 0.97, 95% CI 0.95 to 0.97) water sources were associated with lower prevalence of stunting among children under 5 years old.^43^ In an 8 year retrospective assessment, improved water sources (boreholes, wells, piped, tanks, rainwater and other protected sources) were associated with lower stunting at 1 and 5 years in Ethiopia, but not in Viet Nam, India or Peru.^93^

The difference in results between these studies and ours may lie in the definition of water quality. We characterised water quality using not only drinking water sources, but also the types of water sources used for non-consumption purposes, and the households’ satisfaction with the organoleptic qualities of their main water source (table 1.). In addition, we assessed growth using the continuous LAZ, rather than the dichotomous stunting indicator. Nevertheless, in a combined analysis with more than 500 000 households from 41 LMICs, including Zimbabwe, unimproved water access (water source off-plot) was not associated with HAZ.^49^

Many LMICs have poor water access in that households do not have consistent water supplied directly to their homes,^94^ and often spend more than 30 min for a single water collection round trip.^54^ In our SHINE trial, the time taken by households to fetch water was on average 20 min for a round trip, varying from <5 min to more than 1 hour.^59^ SHINE women reported collecting water less frequently, the longer the time required to fetch water.^64^ Subnational panel data in 59 countries showed that only water piped directly into the home predicted reductions in stunting (β=−0.142; p<0.01),^41^ but not other water sources whether piped off-plot or ground. This may be an indication of the ease of water access. In SSA, a 15 min decrease in water fetching time is associated with improved HAZ (β= +0.3; 95% CI 0.2 to 0.3).^95^ Moreover, in a case–control study in Ethiopia, malnourished children under 5 were more likely than well-nourished children to be from households that had to collect drinking water from sources situated at more than 1 km from the home (OR 4.77; 95% CI 1.01 to 22.71).^96^ In our analyses, where water access was defined by both distance and time to water sources, we found no association with LAZ.

Our hypothesis that WI is related to growth is based on strong theoretical and qualitative studies that suggest pathways from different water dimensions to child growth. Women are usually responsible for water collection in rural LMICs.^97^ Pregnant and postpartum women have stated that the high time and physical burdens associated with water collection compromise their pregnancy and birthing experiences, as well as their postpartum ability to properly care for and feed their children.^48 49 98^ Women in SSA who must collect water outside the home are more likely to leave their young children alone for hours or days at a time.^49^ Women collecting water are also more likely than their male counterparts to experience bodily pain and injuries.^99 100^ Pregnant women having to carry large containers of water can experience physical trauma that affects the development of the fetus. Since growth faltering begins in utero, ensuring water security implies ensuring the safety of the pregnant woman and her offspring. Moreover, such activities leave women with less time and energy to engage in economic or agricultural activities that will ensure adequate diet for themselves and their children.^48^ WI is also disproportionately associated with anxiety and depressive symptoms among women.^85 101–103^ As the primary caregivers of infants and young children, poor maternal physical or mental health can affect growth via poor infant feeding and other caring practices.^48 98 104^ Finally, WI results in dehydration and has been shown to affect lactation among Kenyan women^105^ and breastfeeding practices in other LMICs.^48^ In the Zimbabwean SHINE population, households had approximately 10 L/person of water in the 24 hours prior to their interview,^59^ whereas the WHO recommends a minimum of 50 L/day/person to ensure all basic needs.^106^ For lactating women engaged in even moderate physical activity, drinking water requirement is at least 7.5 L/person.^107^

It is possible that no true association exists between WI and infant growth. However, it is more likely that the association is obscured by complex socioenvironmental confounders and lack of measures to assess these.^108^ Zimbabwean households may be implementing coping behaviours that mitigate the negative health outcomes of WI. Some strategies that have been reported in other water insecure LMICs include: (A) varying water sources based on availability of water bodies above or below ground,^109^ (B) water sharing among neighbours in SSA and in Latin America,^110–112^ (C) modifying water usage to ensure that the available water lasts longer^113^ and (D) boiling or treating water for consumption, although chemical water purification is rare among rural Zimbabwean households.^114^ Truly embracing a broader conceptualisation of household water insecurity that encompass social and behavioural practices for availability, access, quality and utilisation may be necessary to identify pathways towards effective ‘comprehensive’ and ‘transformative’ WASH interventions.

### Strengths and limitations

Our study presents multiple strengths. With increasing occurrence of natural hazard events (eg, droughts, floods, cyclones, hurricanes, windstorms, rising temperatures), population growth and disease outbreaks like the COVID-19 pandemic, the prevalence of growth faltering, WI and FI will worsen in the next few years.^1 54 115–117^ This is predicted to exacerbate all related health and social problems.^117^ Our study is therefore very timely. To our knowledge, this is the first study to investigate the association between growth and growth trajectory by considering FI and WI simultaneously. By using data from a longitudinal cohort, we were able to use repeated measures of infant growth which constitutes an important strength. For anthropometric data, quality control was ensured by repeated training of the nurses who performed data collection.

Based on our literature review, this study is also the first to use multiple indicators of FI and WI in the nutrition sector. The distinction between the FI and WI dimensions increases our understanding of how specific factors are associated with growth faltering in resource-poor settings. For example, instead of solely looking at the problematic food access experiences, we have been able to identify that food availability and food quality are also relevant contributors to infant growth in rural Zimbabwe. Further breakdown of these dimensions may allow specific targets to be set for improving not only infant growth in this population, but also the environment that contributes to poor health and well-being. Using a dimensional approach may help an interdisciplinary approach with both WI and FI, such that intervening on one aspect does not cause negative burdens on the other. This addresses some critical research gaps highlighted by several expert bodies: the Food and Agriculture Organisation,^118^ the Joint Monitoring Programme for Water Supply, Sanitation and Hygiene,^94^ the Development Initiatives,^119^ and various researchers from the food security, water security and nutrition arena.^48 50 120^

Our study is not without limitations. First, the MHFI and MHWI measures were collected when mothers were pregnant. Therefore, we assumed that FI and WI were relatively static, and that FI and WI during pregnancy were valid estimates for the postpartum period up to 18 months. There are two seasons for FI and WI in Zimbabwe: lean (or hungry: January to March) and dry (April to October). The seasons are such that at the time that households are the most food insecure, they are also potentially the least water insecure. Therefore, availability, quality, access and household shocks are likely to be affected during the lean season.^64^ FI during that time is particularly severe for the rural farming communities. This is because the lean period represents low employment opportunities in agriculture and the dwindling of staple food reserves for the household. Similarly, water access, quality and reliability tend to be worse during the dry season.^108 120^ However, in certain instances, due to heavy rains and run-offs water quality may be poor even during the rainy (or wet) season. Future research will need to investigate these seasonal variations in FI and WI on infant growth. This may be done by measuring FI and WI during the postpartum period, ideally at multiple time points so that they can be explored as time-varying predictors of infant growth. Moreover, WI and its dimensions have been strongly associated with FI in LMICs and in fact theorised to drive FI.^50^ Previous publications have reported synergistic effects of FI and WI on health outcomes such as anxiety^121^ and depression.^78 122^ There is also the potential for the mediating effects of FI in the association between WI and health outcomes.^121^ Future studies with adequate power should aim at exploring this association.

The second limitation lies in the exclusion of 35% of mother–infant dyads due to missing observations, potentially resulting in selection bias. The higher percentage of preterm infants and poorer breastfeeding practices within the excluded group may have biased our observed association towards the null since those who were included were likely healthier and more adequately fed. However, in sensitivity analyses adding inverse probability weights to account for missingness yielded results consistent with our main findings (online supplemental table S2). In addition, although we controlled for and tested a large set of covariates, we cannot exclude the probability of unknown confounding, especially for growth trajectory. Some factors that could have led to missingness in the intermediate anthropometric measures lie in the fact that our analyses were based on secondary data. The SHINE trial only aimed at collecting baseline (prior to childbirth) household characteristics and infant data at 18 months. Data at the intermediate time points were only collected when mothers and infants were present during home visits.

## Conclusion

Linear growth, indicated by LAZ, was consistently poorer among infants belonging to households with higher levels of FI, but not WI. Specifically, poor food availability, quality and access were associated with growth faltering from 1 month to 18 months among Zimbabwean infants. There was no evidence of an association between dimensions of FI or WI on infant growth trajectory over time. Our findings support interventions meant to improve food access, availability and quality in rural resource-poor settings. Further research embracing a broader conceptualisation of WI is needed to understand WI in relation to infant growth.

## Data Availability

Data are available on reasonable request. Deidentified participant data are available from the SHINE Trial team and Zvitambo Institute for Maternal and Child Health Research on reasonable request. Please email webadmin@zvitambo.com for requests.

## References

[1] FAO, IFAD, UNICEF, WFP, WHO . The state of food security and nutrition in the world 2020. transforming food systems for affordable healthy diets. Rome: FAO, 2020.

[2] WHO . WHO Multicentre Growth Reference Study Group. In: Who child growth standards: growth velocity based on weight, length and head circumference: methods and development, 2009.

[3] de Onis M , Branca F . Childhood stunting: a global perspective. Matern Child Nutr 2016;12 Suppl 1:12–26. 10.1111/mcn.12231 27187907 PMC5084763

[4] Perumal N , Bassani DG , Roth DE . Use and misuse of stunting as a measure of child health. J Nutr 2018;148:311–5. 10.1093/jn/nxx064 29546307

[5] Prendergast AJ , Humphrey JH . The stunting syndrome in developing countries. Paediatr Int Child Health 2014;34:250–65. 10.1179/2046905514Y.0000000158 25310000 PMC4232245

[6] Leroy JL , Frongillo EA , Dewan P , et al . Can children catch up from the consequences of Undernourishment? Evidence from child linear growth, developmental epigenetics, and brain and neurocognitive development. Adv Nutr 2020;11:1032–41. 10.1093/advances/nmaa020 32584399 PMC7360439

[7] Myatt M , Khara T , Schoenbuchner S , et al . Children who are both wasted and stunted are also underweight and have a high risk of death: a descriptive epidemiology of multiple anthropometric deficits using data from 51 countries. Arch Public Health 2018;76:28. 10.1186/s13690-018-0277-1 30026945 PMC6047117

[8] Black RE , Allen LH , Bhutta ZA , et al . Maternal and child undernutrition: global and regional exposures and health consequences. Lancet 2008;371:243–60. 10.1016/S0140-6736(07)61690-0 18207566

[9] McDonald CM , Olofin I , Flaxman S , et al . The effect of multiple anthropometric deficits on child mortality: meta-analysis of individual data in 10 prospective studies from developing countries. Am J Clin Nutr 2013;97:896–901. 10.3945/ajcn.112.047639 23426036

[10] Walson JL , Berkley JA . The impact of malnutrition on childhood infections. Curr Opin Infect Dis 2018;31:231–6. 10.1097/QCO.0000000000000448 29570495 PMC6037284

[11] Black MM , Walker SP , Fernald LCH , et al . Early childhood development coming of age: science through the life course. Lancet 2017;389:77–90. 10.1016/S0140-6736(16)31389-7 27717614 PMC5884058

[12] de Oliveira KHD , de Almeida GM , Gubert MB , et al . Household food insecurity and early childhood development: systematic review and meta-analysis. Matern Child Nutr 2020;16:e12967. 10.1111/mcn.12967 32052571 PMC7296813

[13] Adair LS , Fall CHD , Osmond C , et al . Associations of linear growth and relative weight gain during early life with adult health and human capital in countries of low and middle income: findings from five birth cohort studies. Lancet 2013;382:525–34. 10.1016/S0140-6736(13)60103-8 23541370 PMC3744751

[14] Asiki G , Newton R , Marions L , et al . The effect of childhood stunting and wasting on adolescent cardiovascular diseases risk and educational achievement in rural Uganda: a retrospective cohort study. Glob Health Action 2019;12:1626184. 10.1080/16549716.2019.1626184 31232215 PMC6598535

[15] Leroy JL , Frongillo EA . Perspective: what does stunting really mean? A critical review of the evidence. Adv Nutr 2019;10:196–204. 10.1093/advances/nmy101 30801614 PMC6416038

[16] UNICEF . Strategy for improved nutrition of children and women in developing countries. New York: United Nations Children’s Fund, 1990. 10.1007/BF02810402 1937618

[17] Bhutta ZA , Akseer N , Keats EC , et al . How countries can reduce child stunting at scale: lessons from exemplar countries. Am J Clin Nutr 2020;112:894S–904. 10.1093/ajcn/nqaa153 32692800 PMC7487427

[18] Bhutta ZA , Das JK , Rizvi A , et al . Evidence-Based interventions for improvement of maternal and child nutrition: what can be done and at what cost? Lancet 2013;382:452–77. 10.1016/S0140-6736(13)60996-4 23746776

[19] Stewart CP , Iannotti L , Dewey KG , et al . Contextualising complementary feeding in a broader framework for stunting prevention. Matern Child Nutr 2013;9 Suppl 2:27–45. 10.1111/mcn.12088 24074316 PMC6860787

[20] IFAD, WFP FAO . The state of food insecurity in the world 2013. The multiple dimensions of food security. Rome, Italy 2013.

[21] Saha KK , Frongillo EA , Alam DS , et al . Household food security is associated with growth of infants and young children in rural Bangladesh. Public Health Nutr 2009;12:1556–62. 10.1017/S1368980009004765 19232147

[22] Mehedi HM , Sayem A , Hoque CMA . Food insecurity and child undernutrition: evidence from BDHS 2011. Journal of Food Security 2013;1.

[23] Gubert MB , Spaniol AM , Bortolini GA , et al . Household food insecurity, nutritional status and morbidity in Brazilian children. Public Health Nutr 2016;19:2240–5. 10.1017/S1368980016000239 26893101 PMC10270926

[24] Betebo B , Ejajo T , Alemseged F , et al . Household food insecurity and its association with nutritional status of children 6-59 months of age in East Badawacho district, South Ethiopia. J Environ Public Health 2017;2017:1–17. 10.1155/2017/6373595 PMC5376409 28408936

[25] Sreeramareddy CT , Ramakrishnareddy N , Subramaniam M . Association between household food access insecurity and nutritional status indicators among children aged <5 years in Nepal: results from a national, cross-sectional household survey. Public Health Nutr 2015;18:2906–14. 10.1017/S1368980014002729 25435296 PMC10271803

[26] Ali D , Saha KK , Nguyen PH , et al . Household food insecurity is associated with higher child undernutrition in Bangladesh, Ethiopia, and Vietnam, but the effect is not mediated by child dietary diversity. J Nutr 2013;143:2015–21. 10.3945/jn.113.175182 24089419

[27] Baig-Ansari N , Rahbar MH , Bhutta ZA , et al . Child's gender and household food insecurity are associated with stunting among young Pakistani children residing in urban squatter settlements. Food Nutr Bull 2006;27:114–27. 10.1177/156482650602700203 16786978

[28] Psaki S , Bhutta ZA , Ahmed T . Household food access and child malnutrition: results from the eight-country MAL-ED study, 2012. 10.1186/1478-7954-10-24 PMC3584951 23237098

[29] Motbainor A , Worku A , Kumie A . Stunting is associated with food diversity while wasting with food insecurity among Underfive children in East and West Gojjam zones of Amhara region, Ethiopia. PLoS One 2015;10:e0133542. 10.1371/journal.pone.0133542 26285047 PMC4540277

[30] Saaka M , Osman SM . Does household food insecurity affect the nutritional status of preschool children aged 6–36 months? Int J Popul Res 2013;2013:304169–12. 10.1155/2013/304169

[31] Osei A , Pandey P , Spiro D , et al . Household food insecurity and nutritional status of children aged 6 to 23 months in Kailali district of Nepal. Food Nutr Bull 2010;31:483–94. 10.1177/156482651003100402

[32] McDonald CM , McLean J , Kroeun H , et al . Household food insecurity and dietary diversity as correlates of maternal and child undernutrition in rural Cambodia. Eur J Clin Nutr 2015;69:242–6. 10.1038/ejcn.2014.161 25117993

[33] Chaparro C . Household food insecurity and nutritional status of women of reproductive age and children under 5 years of age in 5 departments of the Western highlands of Guatemala: an analysis of data from the National maternal-infant health survey 2008-2009 of Guatemala. Washington, DC: Food and Nutrition Technical Assistance III Project (FANTA), 2012.

[34] Humphries DL , Dearden KA , Crookston BT , et al . Cross-Sectional and longitudinal associations between household food security and child anthropometry at ages 5 and 8 years in Ethiopia, India, Peru, and Vietnam. J Nutr 2015;145:1924–33. 10.3945/jn.115.210229 26084361 PMC4516765

[35] Arnold BF , Colford JM . Treating water with chlorine at point-of-use to improve water quality and reduce child diarrhea in developing countries: a systematic review and meta-analysis. Am J Trop Med Hyg 2007;76:354–64. 10.4269/ajtmh.2007.76.354 17297049

[36] Mattioli MC , Boehm AB , Davis J , et al . Enteric pathogens in stored drinking water and on caregiver's hands in Tanzanian households with and without reported cases of child diarrhea. PLoS One 2014;9:e84939. 10.1371/journal.pone.0084939 24392161 PMC3879350

[37] Nounkeu C , Kamgno J , Dharod J . Assessment of the relationship between water insecurity, hygiene practices, and incidence of diarrhea among children from rural households of the Menoua division, West Cameroon. J Public Health Afr 2019;10:951. 10.4081/jphia.2019.951 31285814 PMC6589635

[38] Shrestha SK , Vicendese D , Erbas B . Water, sanitation and hygiene practices associated with improved height-for-age, weight-for-height and weight-for-age z-scores among under-five children in Nepal. BMC Pediatr 2020;20:134. 10.1186/s12887-020-2010-9 32293376 PMC7092611

[39] Luby SP , Rahman M , Arnold BF , et al . Effects of water quality, sanitation, handwashing, and nutritional interventions on diarrhoea and child growth in rural Bangladesh: a cluster randomised controlled trial. Lancet Glob Health 2018;6:e302–15. 10.1016/S2214-109X(17)30490-4 29396217 PMC5809718

[40] Null C , Stewart CP , Pickering AJ , et al . Effects of water quality, sanitation, handwashing, and nutritional interventions on diarrhoea and child growth in rural Kenya: a cluster-randomised controlled trial. Lancet Glob Health 2018;6:e316–29. 10.1016/S2214-109X(18)30005-6 29396219 PMC5809717

[41] Headey D , Palloni G , Water PG . Water, sanitation, and child health: evidence from Subnational panel data in 59 countries. Demography 2019;56:729–52. 10.1007/s13524-019-00760-y 30820757 PMC6449314

[42] Chirande L , Charwe D , Mbwana H , et al . Determinants of stunting and severe stunting among under-fives in Tanzania: evidence from the 2010 cross-sectional household survey. BMC Pediatr 2015;15:165. 10.1186/s12887-015-0482-9 26489405 PMC4618754

[43] Fink G , Günther I , Hill K . The effect of water and sanitation on child health: evidence from the demographic and health surveys 1986-2007. Int J Epidemiol 2011;40:1196–204. 10.1093/ije/dyr102 21724576

[44] Merchant AT , Jones C , Kiure A , et al . Water and sanitation associated with improved child growth. Eur J Clin Nutr 2003;57:1562–8. 10.1038/sj.ejcn.1601725 14647221

[45] HWISE . Household water insecurity experiences scale. A cross-culturally validated scale to measure water insecurity at household level, 2019. Available: https://sites.northwestern.edu/hwise/

[46] Young SL , Boateng GO , Jamaluddine Z , et al . The household water insecurity experiences (HWISE) scale: development and validation of a household water insecurity measure for low-income and middle-income countries. BMJ Glob Health 2019;4:e001750. 10.1136/bmjgh-2019-001750 PMC6768340 31637027

[47] Jepson W , Budds J , Eichelberger L , et al . Advancing human capabilities for water security: a relational approach. Water Secur 2017;1:46–52. 10.1016/j.wasec.2017.07.001 PMC5844501 29532811

[48] Schuster RC , Butler MS , Wutich A . Household Water Insecurity Experiences-Research Coordination N. “If there is no water, we cannot feed our children”: The far-reaching consequences of water insecurity on infant feeding practices and infant health across 16 low- and middle-income countries. American Journal of Human Biology 2019;32:e23357. 31868269 10.1002/ajhb.23357 PMC7537364

[49] Geere J-AL , Hunter PR . The association of water carriage, water supply and sanitation usage with maternal and child health. A combined analysis of 49 multiple indicator cluster surveys from 41 countries. Int J Hyg Environ Health 2020;223:238–47. 10.1016/j.ijheh.2019.08.007 31488359

[50] Brewis A , Workman C , Wutich A . Household Water Insecurity Experiences - Research Coordination N. Household water insecurity is strongly associated with food insecurity: Evidence from 27 sites in low- and middle-income countries. Am J Hum Biol 2019:e23309. 31444940 10.1002/ajhb.23309 PMC9942689

[51] Choudhary N , Schuster R , Brewis A , et al . Water insecurity potentially undermines dietary diversity of children aged 6-23 months: evidence from India. Matern Child Nutr 2020;16:e12929. 10.1111/mcn.12929 31999395 PMC7083507

[52] Choudhary N , Schuster RC , Brewis A , et al . Household water insecurity affects child nutrition through alternative pathways to wash: evidence from India. Food Nutr Bull 2021;42:170–87. 10.1177/0379572121998122 34282660

[53] Victora CG , Christian P , Vidaletti LP , et al . Revisiting maternal and child undernutrition in low-income and middle-income countries: variable progress towards an unfinished agenda. Lancet 2021;397:1388–99. 10.1016/S0140-6736(21)00394-9 33691094 PMC7613170

[54] Drinking Water WHO , 2019. Available: https://www.who.int/news-room/fact-sheets/detail/drinking-water [Accessed 7 Apr 2021].

[55] ZimStat . Inter-censal demographic 2017, 2017.

[56] ZimVAC . Rural Livelihoods assessment report. Zimbabwe: Zimbabwe Vulnerability Assessement Committee, 2019.

[57] Gough EK , Moodie EE , Prendergast AJ , et al . Linear growth trajectories in Zimbabwean infants. Am J Clin Nutr 2016;104:1616–27. 10.3945/ajcn.116.133538 27806980 PMC5118730

[58] Jones AD , Rukobo S , Chasekwa B , et al . Acute illness is associated with suppression of the growth hormone axis in Zimbabwean infants. Am J Trop Med Hyg 2015;92:463–70. 10.4269/ajtmh.14-0448 25535308 PMC4347356

[59] Humphrey JH , Mbuya MNN , Ntozini R , et al . Independent and combined effects of improved water, sanitation, and hygiene, and improved complementary feeding, on child stunting and anaemia in rural Zimbabwe: a cluster-randomised trial. Lancet Glob Health 2019;7:e132–47. 10.1016/S2214-109X(18)30374-7 30554749 PMC6293965

[60] EC . Zimbabwe: country profile on nutrition and child stunting trends, 2019. Available: https://ec.europa.eu/europeaid/zimbabwe-nutrition-country-fiche-and-child-stunting-trends_en

[61] UNICEF . IYCF programming guide, 2011.

[62] , Humphrey JH , Jones AD , et al, Sanitation Hygiene Infant Nutrition Efficacy (SHINE) Trial Team . The sanitation hygiene infant nutrition efficacy (shine) trial: rationale, design, and methods. Clin Infect Dis 2015;61 Suppl 7:S685–702. 10.1093/cid/civ844 26602296 PMC4657589

[63] WHO child growth standards : length/height-for-age, weight-for-age, weight-for-length, weight -for-height and body mass index-for-age : methods and development. 2006.

[64] Koyratty N , Jones AD , Schuster R , et al . Food insecurity and water insecurity in rural Zimbabwe: development of multidimensional household measures. Int J Environ Res Public Health 2021;18. doi:10.3390/ijerph18116020. [Epub ahead of print: 03 06 2021]. PMC8199942 34205143

[65] Chasekwa B , Maluccio JA , Ntozini R . Measuring wealth in rural communities: lessons from the sanitation, hygiene. Infant Nutrition Efficacy (SHINE) trial 2018. 10.1371/journal.pone.0199393 PMC6023145 29953495

[66] Chibanda D , Mangezi W , Tshimanga M , et al . Validation of the Edinburgh postnatal depression scale among women in a high HIV prevalence area in urban Zimbabwe. Arch Womens Ment Health 2010;13:201–6. 10.1007/s00737-009-0073-6 19760051

[67] Matare CR , Mbuya MNN , Dickin KL . Maternal capabilities are associated with child caregiving behaviors among women in rural Zimbabwe. J Nutr 2020. 10.1093/jn/nxaa255 PMC7948208 33211881

[68] FAO 360. F . Minimum dietary diversity for women: a guide for measurement. Rome: FAO, 2016.

[69] Ali NB , Tahsina T , Hoque DME , et al . Association of food security and other socio-economic factors with dietary diversity and nutritional statuses of children aged 6-59 months in rural Bangladesh. PLoS One 2019;14:e0221929. 10.1371/journal.pone.0221929 31465509 PMC6715227

[70] Leroy JL , Ruel M , Habicht J-P , et al . Linear growth deficit continues to accumulate beyond the first 1000 days in low- and middle-income countries: global evidence from 51 national surveys. J Nutr 2014;144:1460–6. 10.3945/jn.114.191981 24944283

[71] Smith LE . Convergence and divergence in statistical and programmatic approaches to address child stunting and wasting. J Nutr 2018;148:823–4. 10.1093/jn/nxy098 29878270

[72] Ruel MT , Quisumbing AR , Balagamwala M . Nutrition-sensitive agriculture: what have we learned so far? Glob Food Sec 2018;17:128–53. 10.1016/j.gfs.2018.01.002

[73] Kavle JA , Landry M . Addressing barriers to maternal nutrition in low- and middle-income countries: a review of the evidence and programme implications. Matern Child Nutr 2018;14:e12508. 10.1111/mcn.12508 28836343 PMC5763330

[74] Young SL , Plenty AHJ , Luwedde FA , et al . Household food insecurity, maternal nutritional status, and infant feeding practices among HIV-infected Ugandan women receiving combination antiretroviral therapy. Matern Child Health J 2014;18:2044–53. 10.1007/s10995-014-1450-y 24585398 PMC4419705

[75] Maitra C , Sethi V , Unisa S , et al . Household food insecurity and maternal and child nutritional status: evidence from Maharashtra. Rev Income Wealth 2019;65. 10.1111/roiw.12436

[76] Huddleston-Casas C , Charnigo R , Simmons LA . Food insecurity and maternal depression in rural, low-income families: a longitudinal investigation. Public Health Nutr 2009;12:1133–40. 10.1017/S1368980008003650 18789167

[77] Leung CW , Epel ES , Willett WC , et al . Household food insecurity is positively associated with depression among low-income supplemental nutrition assistance program participants and Income-Eligible Nonparticipants. J Nutr 2015;145:622–7. 10.3945/jn.114.199414 25733480

[78] Boateng GO , Workman CL , Miller JD , et al . The syndemic effects of food insecurity, water insecurity, and HIV on depressive symptomatology among Kenyan women. Soc Sci Med 2022;295:113043. 10.1016/j.socscimed.2020.113043 32482382 PMC8869838

[79] Tuthill EL , Maltby A , Conteh J , et al . Persistent food insecurity, but not HIV, is associated with depressive symptoms among perinatal women in Kenya: a longitudinal perspective. AIDS Behav 2021;25:847–55. 10.1007/s10461-020-03047-1 32989575 PMC7886965

[80] Matare CR , Mbuya MN , Pelto G . Sanitation hygiene infant nutrition efficacy trial T. assessing maternal capabilities in the shine trial: highlighting a hidden link in the causal pathway to child health. Clin Infect Dis 2015;61:S745–51. 26602303 10.1093/cid/civ851 PMC4657596

[81] Awungafac G , Mugamba S , Nalugoda F , et al . Household food insecurity and its association with self-reported male perpetration of intimate partner violence: a survey of two districts in central and Western Uganda. BMJ Open 2021;11:e045427. 10.1136/bmjopen-2020-045427 PMC8016075 33789856

[82] Diamond-Smith N , Conroy AA , Tsai AC , et al . Food insecurity and intimate partner violence among married women in Nepal. J Glob Health 2019;9::010412–10412. 10.7189/jogh.09.010412 PMC6359930 30774941

[83] Hurley KM , Black MM , Papas MA , et al . Maternal symptoms of stress, depression, and anxiety are related to nonresponsive feeding styles in a statewide sample of WIC participants. J Nutr 2008;138:799–805. 10.1093/jn/138.4.799 18356338 PMC3137941

[84] Wachs TD , Black MM , Engle PL . Maternal Depression: A Global Threat to Children’s Health, Development, and Behavior and to Human Rights. Child Dev Perspect 2009;3:51–9. 10.1111/j.1750-8606.2008.00077.x

[85] Zubaran C , Foresti K . The correlation between breastfeeding self-efficacy and maternal postpartum depression in southern Brazil. Sex Reprod Healthc 2013;4:9–15. 10.1016/j.srhc.2012.12.001 23427927

[86] Saha KK , Frongillo EA , Alam DS , et al . Household food security is associated with infant feeding practices in rural Bangladesh. J Nutr 2008;138:1383–90. 10.1093/jn/138.7.1383 18567765 PMC2518644

[87] Bronte-Tinkew J , Zaslow M , Capps R , et al . Food insecurity works through depression, parenting, and infant feeding to influence overweight and health in toddlers. J Nutr 2007;137:2160–5. 10.1093/jn/137.9.2160 17709458

[88] Surkan PJ , Kennedy CE , Hurley KM , et al . Maternal depression and early childhood growth in developing countries: systematic review and meta-analysis. Bull World Health Organ 2011;89:608–15. 10.2471/BLT.11.088187 21836759 PMC3150769

[89] Chai J , Fink G , Kaaya S , et al . Association between intimate partner violence and poor child growth: results from 42 demographic and health surveys. Bull World Health Organ 2016;94:331–9. 10.2471/BLT.15.152462 27147763 PMC4850526

[90] Maitra C . A review of studies examining the link between food insecurity and malnutrition. Technical Paper. Rome 2018.

[91] Chandrasekhar S , Aguayo VM , Krishna V , et al . Household food insecurity and children's dietary diversity and nutrition in India. Evidence from the comprehensive nutrition survey in Maharashtra. Matern Child Nutr 2017;13 Suppl 2:e12447. 10.1111/mcn.12447 29032621 PMC6866156

[92] Checkley W , Buckley G , Gilman RH , et al . Multi-country analysis of the effects of diarrhoea on childhood stunting. Int J Epidemiol 2008;37:816–30. 10.1093/ije/dyn099 18567626 PMC2734063

[93] Dearden KA , Schott W , Crookston BT , et al . Children with access to improved sanitation but not improved water are at lower risk of stunting compared to children without access: a cohort study in Ethiopia, India, Peru, and Vietnam. BMC Public Health 2017;17:110. 10.1186/s12889-017-4033-1 28114914 PMC5259877

[94] WHO/UNICEF joint monitoring programme for water supply, sanitation and hygiene (JMP), 2019. Available: https://washdata.org/how-we-work/about-jmp

[95] Pickering AJ , Null C , Winch PJ , et al . The wash benefits and shine trials: interpretation of wash intervention effects on linear growth and diarrhoea. Lancet Glob Health 2019;7:e1139–46. 10.1016/S2214-109X(19)30268-2 31303300

[96] Soboksa NE , Gari SR , Hailu AB , et al . Childhood malnutrition and the association with diarrhea, water supply, sanitation, and hygiene practices in Kersa and Omo NADA districts of Jimma zone, Ethiopia. Environ Health Insights 2021;15:1178630221999635. 10.1177/1178630221999635 33746513 PMC7940723

[97] Graham JP , Hirai M , Kim S-S . An analysis of water collection labor among women and children in 24 sub-Saharan African countries. PLoS One 2016;11:e0155981. 10.1371/journal.pone.0155981 27248494 PMC4889070

[98] Collins SM , Mbullo Owuor P , Miller JD , et al . 'I know how stressful it is to lack water!' Exploring the lived experiences of household water insecurity among pregnant and postpartum women in western Kenya. Glob Public Health 2019;14:649–62. 10.1080/17441692.2018.1521861 30231793 PMC6414268

[99] Venkataramanan V , Geere J-AL , Thomae B , et al . In pursuit of 'safe' water: the burden of personal injury from water fetching in 21 low-income and middle-income countries. BMJ Glob Health 2020;5:e003328. 10.1136/bmjgh-2020-003328 PMC7592242 33115862

[100] Geere J-AL , Hunter PR , Jagals P . Domestic water carrying and its implications for health: a review and mixed methods pilot study in Limpopo Province, South Africa. Environ Health 2010;9:52. 10.1186/1476-069X-9-52 20796292 PMC2939590

[101] Brewis A , Choudhary N , Wutich A . Low water access as a gendered physiological stressor: blood pressure evidence from Nepal. Am J Hum Biol 2019;31:e23234. 10.1002/ajhb.23234 30900309

[102] Cooper-Vince CE , Arachy H , Kakuhikire B , et al . Water insecurity and gendered risk for depression in rural Uganda: a hotspot analysis. BMC Public Health 2018;18:1143. 10.1186/s12889-018-6043-z 30257659 PMC6158871

[103] Tallman PS . Water insecurity and mental health in the Amazon: economic and ecological drivers of distress. Econ Anthropol 2019;6:304–16. 10.1002/sea2.12144

[104] Cooper-Vince CE , Kakuhikire B , Vorechovska D , et al . Household water insecurity, missed schooling, and the mediating role of caregiver depression in rural Uganda. Glob Ment Health 2017;4:e15. 10.1017/gmh.2017.14 PMC5719478 29230311

[105] Bethancourt HJ , Swanson ZS , Nzunza R , et al . Hydration in relation to water insecurity, heat index, and lactation status in two small-scale populations in hot-humid and hot-arid environments. Am J Hum Biol 2021;33:e23447. 10.1002/ajhb.23447 32583580 PMC8829588

[106] WHO . The human right to water and sanitation. Media Brief 2010.

[107] UNDP . Human development report 2006: beyond scarcity: power, poverty and the global water crisis. UNDP 2006.

[108] Smiley SL , Stoler J . Socio‐environmental confounders of safe water interventions. WIREs Water 2020;7:e1438. 10.1002/wat2.1438

[109] Shaheed A , Orgill J , Ratana C , et al . Water quality risks of 'improved' water sources: evidence from Cambodia. Trop Med Int Health 2014;19:186–94. 10.1111/tmi.12229 24252094

[110] Stoler J , Brewis A , Harris LM , et al . Household water sharing: a missing link in international health. Int Health 2019;11:163–5. 10.1093/inthealth/ihy094 30576501 PMC6484635

[111] Wutich A , Budds J , Jepson W . Household water sharing: a review of water gifts, exchanges, and transfers across cultures. Wiley Interdisciplinary Reviews: Water, 2018. 10.1002/wat2.1309 PMC6407694 30858971

[112] Brewis A , Rosinger A , Wutich A . Water sharing, reciprocity, and need: a comparative study of interhousehold water transfers in sub-Saharan Africa. Econ Anthropol 2019;6:208–21. 10.1002/sea2.12143

[113] Wutich A , Ragsdale K . Water insecurity and emotional distress: coping with supply, access, and seasonal variability of water in a Bolivian squatter settlement. Soc Sci Med 2008;67:2116–25. 10.1016/j.socscimed.2008.09.042 18954928

[114] ZimStat ICF . Zimbabwe Demographic and Health Survey 2015: Final Report. In: Zimbabwe national statistics agency, 2015.

[115] Stoler J , Miller JD , Brewis A , et al . Household water insecurity will complicate the ongoing COVID-19 response: evidence from 29 sites in 23 low- and middle-income countries. Int J Hyg Environ Health 2021;234:113715. 10.1016/j.ijheh.2021.113715 33735823 PMC7894133

[116] IFRC . World disasters report 2020. come heat or high water. Geneva: International Federation of Red Cross and Red Crescent Societies, 2020.

[117] FSIN . Global report on food crises. joint analysis for better decisions. Food Security Information Network 2020.

[118] FAO . Proceedings International Scientific Symposium on Food and Nutrition Security information: From Valid Measurement to Effective Decision Making. In: Paper presented at: international scientific Symposium on food and nutrition security. Rome, Italy, 2012.

[119] Global Nutrition Report: Action on equity to end malnutrition. 2020. Bristol, UK.

[120] Young SL , Frongillo EA , Jamaluddine Z . Perspective: the importance of water security for ensuring food security, good nutrition, and well-being. Advances in nutrition 2021. 10.1093/advances/nmab003 PMC8321834 33601407

[121] Brewis A , Choudhary N , Wutich A . Household water insecurity may influence common mental disorders directly and indirectly through multiple pathways: evidence from Haiti. Soc Sci Med 2019;238:112520. 10.1016/j.socscimed.2019.112520 31473576

[122] Workman CL , Ureksoy H . Water insecurity in a syndemic context: understanding the psycho-emotional stress of water insecurity in Lesotho, Africa. Soc Sci Med 2017;179:52–60. 10.1016/j.socscimed.2017.02.026 28254659

