# Supplementary material for: Growth and growth trajectory among infants in early life: contributions of food insecurity and water insecurity in rural Zimbabwe
Source: BMJ Nutr Prev Health. 2022 Nov 30;5(2):332–43. doi: 10.1136/bmjnph-2022-000470 (PMC9813639; doi:10.1136/bmjnph-2022-000470)
Supplement: Supplementary data [file bmjnph-2022-000470supp001.pdf]

Supplemental Materials

Table S1. Association of infant length and length trajectory with food insecurity and water insecurity

	M1-M18 (n=714)		M6-M18 (n=710)	
**Models**	**MAM**	**FAM**	**MAN**	**FAM**
**Length (cm)**				
**Food Insecurity**				
**Poor Food Access**	-0.16 (-0.41, 0.1)	-0.13 (-0.36, 0.1)	-0.13 (-0.39, 0.12)	-0.13 (-0.36, 0.11)
**Household Shocks**	-0.1 (-0.35, 0.15)	-0.05 (-0.29, 0.18)	0.13 (-0.12, 0.38)	0.17 (-0.07, 0.4)
**Low Food availability & Quality**	-0.24 (-0.49, 0.02)	**-0.26 (-0.50, -0.02)**	0.05 (-0.2, 0.31)	0.05 (-0.19, 0.29)
**Water Insecurity**				
**Poor Water Access**	-0.13 (-0.38, 0.12)	-0.11 (-0.34, 0.12)	-0.1 (-0.35, 0.16)	-0.12 (-0.35, 0.12)
**Poor Water Quality**	0.02 (-0.22, 0.26)	-0.05 (-0.28, 0.17)	-0.06 (-0.3, 0.18)	-0.12 (-0.35, 0.1)
**Low Water Reliability**	-0.05 (-0.3, 0.21)	-0.07 (-0.3, 0.16)	0.01 (-0.25, 0.26)	-0.03 (-0.26, 0.21)
**Length change (cm)**				
**Time**	**1.29 (1.27, 1.32)**	0.36 (-0.11, 0.83)	**0.93 (0.91, 0.96)**	0.19 (-0.31, 0.7)
**Time x Food Insecurity**				
**Poor Food Access**	0 (-0.02, 0.02)	0 (-0.02, 0.02)	0 (-0.02, 0.02)	0 (-0.03, 0.02)
**Household Shocks**	0.02 (0, 0.04)	0.02 (0, 0.04)	0 (-0.02, 0.02)	0 (-0.02, 0.02)
**Low Food availability & Quality**	0 (-0.02, 0.02)	0 (-0.02, 0.02)	**-0.03 (-0.05, -0.01)**	-0.02 (-0.04, 0)
**Time x Water Insecurity**				
**Poor Water Access**	0.01 (-0.01, 0.03)	0 (-0.01, 0.02)	0.01 (-0.01, 0.03)	0.01 (-0.01, 0.03)
**Poor Water Quality**	0.01 (-0.01, 0.03)	0.01 (-0.01, 0.03)	0.02 (0, 0.04)	0.02 (0, 0.04)
**Low Water Reliability**	0 (-0.02, 0.02)	0.01 (-0.01, 0.02)	0 (-0.02, 0.02)	0 (-0.02, 0.03)

MAM=Minimally-adjusted model: season at baseline interview (calendar quarter), SES (tertile), infant sex (female vs. male), household location (Chirumanzu vs. Shurugwi), improved latrine (no vs. yes), and maternal pregnancy HIV status (positive vs. negative), time-sex interaction.

FAM=Fully-adjusted model: MAM + maternal age (years), height (cm), education (some primary, some secondary, completed secondary), religion (Apostolic, Other Christian, Other religion), infant birthweight (kg), preterm (born at <37 weeks of gestation: yes vs. no), household size (number of members), continued breastfeeding until M18 (yes vs. no), and time interactions with maternal height, infant birthweight and continued breastfeeding until M18.

Table S2. Association of infant LAZ and LAZ trajectory with food insecurity and water insecurity weighted for missingness

	M1-M18		M6-M18	
**Models**	**MAM**	**FAM**	**MAN**	**FAM**
**LAZ**				
**Food Insecurity**				
**Poor Food Access**	-0.05 [-0.15, 0.05]	-0.03 [-0.1, 0.05]	**-0.13 [-0.22, -0.03]**	**-0.11 [-0.2, -0.03]**
**Household Shocks**	-0.02 [-0.12, 0.07]	-0.02 [-0.09, 0.06]	-0.01 [-0.1, 0.08]	0 [-0.08, 0.09]
**Low Food availability & Quality**	-0.09 [-0.19, 0.01]	**-0.09 [-0.17, -0.01]**	-0.08 [-0.18, 0.02]	-0.06 [-0.14, 0.03]
**Water Insecurity**				
**Poor Water Access**	-0.01 [-0.11, 0.08]	0 [-0.08, 0.07]	-0.02 [-0.12, 0.08]	-0.02 [-0.1, 0.07]
**Poor Water Quality**	0.04 [-0.05, 0.14]	0 [-0.08, 0.07]	0.08 [-0.01, 0.17]	0.04 [-0.04, 0.12]
**Low Water Reliability**	0.05 [-0.04, 0.15]	0.03 [-0.05, 0.1]	0.05 [-0.04, 0.14]	0.03 [-0.05, 0.12]
**LAZ Trajectory**				
**Time**	**-0.03 [-0.04, -0.03]**	**-0.21 [-0.33, -0.09]**	**-0.04 [-0.06, -0.03]**	-0.13 [-0.28, 0.03]
**Time x Food Insecurity**				
**Poor Food Access**	0 [-0.01, 0]	0 [-0.01, 0]	0 [0, 0.01]	0 [0, 0.01]
**Household Shocks**	0 [0, 0.01]	0 [0, 0.01]	0 [0, 0.01]	0 [0, 0.01]
**Low Food availability & Quality**	0 [-0.01, 0]	0 [-0.01, 0]	0 [-0.01, 0]	0 [-0.01, 0]
**Time x Water Insecurity**				
**Poor Water Access**	0 [-0.01, 0]	0 [-0.01, 0]	0 [-0.01, 0.01]	0 [-0.01, 0]
**Poor Water Quality**	0 [0, 0.01]	0 [0, 0.01]	0 [-0.01, 0.01]	0 [-0.01, 0.01]
**Low Water Reliability**	0 [-0.01, 0]	0 [-0.01, 0]	0 [-0.01, 0]	0 [-0.01, 0]

MAM=Minimally-adjusted model: season at baseline interview (calendar quarter), SES (tertile), infant sex (female vs. male), household location (Chirumanzu vs. Shurugwi), improved latrine (no vs. yes), and maternal pregnancy HIV status (positive vs. negative), time-sex interaction.

FAM=Fully-adjusted model: MAM + maternal age (years), height (cm), education (some primary, some secondary, completed secondary), religion (Apostolic, Other Christian, Other religion), infant birthweight (kg), preterm (born at <37 weeks of gestation: yes vs. no), household size (number of members), continued breastfeeding until M18 (yes vs. no), and time interactions with maternal height, infant birthweight and continued breastfeeding until M18.
